# Mobilität älterer Menschen im öffentlichen Raum unter Gendergesichtspunkten

**DOI:** 10.1007/s00103-024-03928-z

**Published:** 2024-07-16

**Authors:** Gisela Stete, Brigitte Wotha

**Affiliations:** 1 StetePlanung, Büro für Stadt- und Verkehrsplanung, Darmstadt, Deutschland; 2 Arbeitskreis „Gender und Mobilität“ der Forschungsgesellschaft für Straßen- und Verkehrswesen e. V., Köln, Deutschland; 3 Fachhochschule Kiel, Moorblöcken 12, 24149 Kiel, Deutschland

**Keywords:** Gendersensible Mobilität, Mobilitätsbedarfe, Mobilitätschancen, Wegeketten, Alltagsmobilität, Gender-sensitive mobility, Mobility needs, Mobility opportunities, Trip chains, Everyday mobility

## Abstract

Für Menschen im höheren Lebensalter ist ein abgestimmtes Mobilitätsangebot unerlässliche Voraussetzung für die Teilhabe am gesellschaftlichen Leben und für mehr Lebensqualität. Das Ziel sollte eine selbstbestimmte Alltagsmobilität sein, die ein zufriedenstellendes und gesundes Leben im Alter ermöglicht. Mobilität ist von den vielfältigen individuellen Fähigkeiten und Bedürfnissen der einzelnen Menschen abhängig. Demgegenüber begrenzen gesellschaftliche Strukturen, regulatorische und räumliche Rahmenbedingungen die Umsetzung der Mobilitätsangebote und ihrer verkehrlichen Infrastruktur. Diese Rahmenbedingungen werden von der Wahrnehmung sozialer Rollen, der Verfügbarkeit von Ressourcen und geschlechtsspezifischen Zuschreibungen geprägt. So liegt z. B. bei über 50-Jährigen die Pflege- und Sorgearbeit überwiegend bei den Frauen. Das Angebot an Verkehrsinfrastruktur richtet sich aktuell an Zielen aus, die von den Bedarfen der Erwerbsarbeit und nicht der Versorgungsarbeit geprägt sind. Umgekehrt nimmt die Verfügbarkeit flexibler Verkehrsmittel, wie z. B. Autos, bei Frauen im höheren Alter auch aus ökonomischen Gründen ab. Daher sollten bei der Mobilitätsplanung verstärkt die spezifischen Bedürfnisse, wie z. B. die Ausrichtung auf Ziele der Versorgung, Beratung und Gesundheitsangebote, sowie eine größere Nahraumorientierung berücksichtigt werden. Insbesondere für ältere Frauen sind Sicherheitsbedürfnisse oftmals ein wichtiger Entscheidungsfaktor, mobil zu werden oder auf Mobilität zu verzichten. Das beinhaltet sowohl die gefühlte subjektive Sicherheit, die Barrierefreiheit als auch den Schutz vor gefährdenden Witterungsbedingungen wie einem glatten Untergrund oder übermäßiger Hitzeexposition. Bei Berücksichtigung dieser Grundlagen kann für mehr Menschen eine selbstbestimmte Teilhabe am gesellschaftlichen Leben gewährleistet werden.

## Abstract (en)

For older people, a sufficient mobility offer is an essential prerequisite for participation in social life and a better quality of life. The goal should be self-determined everyday mobility that enables a satisfying and healthy life. Mobility depends on diverse individual abilities and needs. On the other hand, social structures as well as regulatory and spatial framework conditions limit the realisation of mobility services and their transport infrastructure. These framework conditions are characterised by the perception of social roles, the availability of resources and gender-specific attributions. For example, care and caring work is predominantly the responsibility of women in the age group of 50 years plus. The availability of transport infrastructure is geared towards goals that are characterised by the needs of gainful employment and not the goals of care work. Conversely, the availability of flexible means of transport, such as cars, also decreases for women in old age for economic reasons. For this reason, mobility planning should take greater account of specific needs, such as orientation towards care objectives, counselling, and health services and place a greater focus on the local area. For elderly people, especially women, security needs are often an important factor in the decision to become mobile or to forego mobility. This includes perceived subjective safety, accessibility and protection from weather conditions such as slippery surfaces or exposure to excessive heat. If these basic principles are taken into account, more people can be guaranteed self-determined participation in social life.

## Mobilität als Grundlage für Teilhabe und Lebensqualität im Alter

Mobilität ist die Voraussetzung für Teilhabe am sozialen, wirtschaftlichen und politischen Leben in unserer Gesellschaft und essentielle Grundlage für Lebensqualität im Alter. Mit Mobilität ist die Fähigkeit gemeint, verschiedene Ziele für gewünschte Zwecke in einer bestimmten Zeit zu erreichen, um Aktivitäten auszuüben und Bedürfnisse zu befriedigen [1]. Demgegenüber ist Verkehr die realisierte Ortsveränderung zwischen verschiedenen Räumen. Mobilität an sich ist nicht an ein bestimmtes Verkehrsmittel gebunden. Mobil sein können Menschen zu Fuß, mit dem Fahrrad, mit dem Öffentlichen Personennahverkehr (ÖPNV, schienen- oder straßengebunden) oder dem motorisierten Individualverkehr (selbst fahrend, mitfahrend im Kraftfahrzeug oder z. B. Elektroroller) – abhängig von ihren vielfältigen individuellen Fähigkeiten und Bedürfnissen.

Die qualitativen Kennwerte von Mobilität werden beschrieben mit:

- Mobilitätsbedarf: Warum will/muss (Zweck) ich wohin (Ziel), um Dinge zu erledigen? Und:
- Mobilitätschancen: Wie erreiche ich wann und unter welchen Bedingungen meine Ziele?

Die Mobilität im Alter ist über abnehmende individuelle körperliche oder kognitive Veränderungen hinaus von verschiedenen strukturellen, infrastrukturellen und sozioökonomischen Faktoren beeinflusst. Diese Faktoren korrelieren oftmals mit einer geschlechtlichen Determinante (s. unten). Mobilitätsbedarf und Mobilitätschancen sind zudem abhängig von sozialen Rollen und Zuständigkeiten in unserer Gesellschaft. Mobilität unter Genderaspekten zu betrachten bedeutet, die unterschiedlichen sozialen Rollenzuschreibungen für Frauen und Männer^1^ in unserer Gesellschaft wahrzunehmen. Diese Rollenzuschreibungen beinhalten Erwerbstätigkeit, Erziehungsarbeit, Hausarbeit, Versorgungsarbeit und Betreuungsarbeit und stehen oftmals in enger Wechselwirkung mit der Familiensituation, z. B. Haushalt mit und ohne Kinder, Jugendliche und/oder ältere, z. T. betreuungsbedürftige Familienangehörige. Im Lebensalltag der Menschen sind diese Rollen unter den Geschlechtern so verteilt, dass Frauen nachweislich den höheren Anteil an der Familienarbeit leisten, und zwar unabhängig davon, ob sie erwerbstätig sind oder nicht [2].

Die Ausprägungen dieser Rollenzuschreibungen setzen sich auch im höheren Alter fort. Insbesondere die informelle Pflege von Familienangehörigen wird in Deutschland überwiegend von Frauen geleistet. So liegt der Gender Care Gap für Menschen älter als 50 Jahre, der die informelle Pflege *außerhalb* des eigenen Haushalts beschreibt, in Deutschland bei 113 %. Das heißt, dass Frauen mehr als doppelt so häufig auch außerhalb des eigenen Haushalts pflegen wie Männer [3]. Aus diesen Rollenzuschreibungen entstehen spezifische Mobilitätsbedarfe, die in den bestehenden Verkehrsdatenerhebungen nur bedingt berücksichtigt werden und damit für die Planungen von z. B. Ausgestaltung von Verkehrsmitteln, Verkehrswegen oder Routenplanungen nicht oder wenig zur Verfügung stehen.

Die Gendersicht auf Mobilität beinhaltet nicht nur die Perspektive auf die einzelnen Individuen, sondern auch die Sicht auf die gesellschaftlichen Strukturen und institutionellen sowie räumlichen Rahmenbedingungen, die die Rollen und ihre Ausgestaltung prägen und verstärken. Mobilität wird damit durch personenbezogene und umweltbezogene Determinanten bestimmt [1]. Dies wird dann deutlich, wenn Räume und Siedlungsentwicklung mit geschlechtsspezifischen Rollenzuschreibungen und Nutzungsmustern verknüpft werden oder umgekehrt die Berücksichtigung von Care-Aufgaben fehlt [4].

**Abb. 1:**
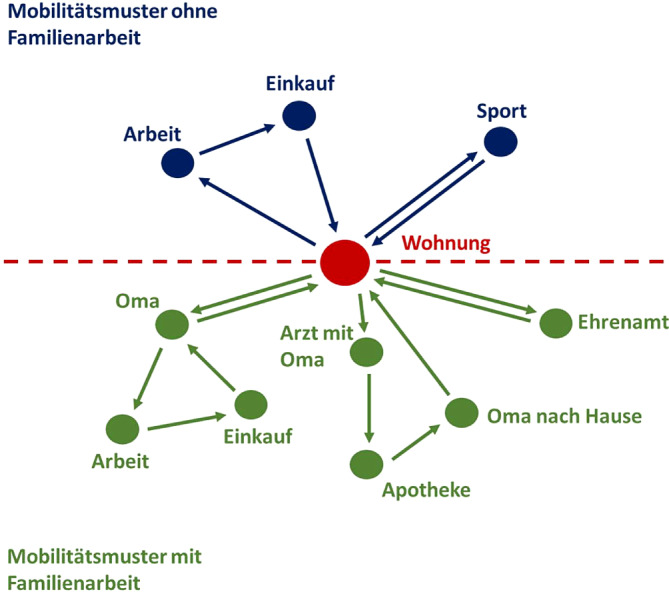
Mobilitätsmuster mit und ohne Familienarbeit – Wegeketten (Quelle: eigene Abbildung von Gisela Stete)

Ein wesentliches Merkmal von Menschen, die Erwerbs‑, Familien- und Versorgungstätigkeiten miteinander verbinden, ist die Bildung von Wegeketten. Eine Wegekette besteht aus der zeitlichen Abfolge mehrerer Wege zur Erledigung von Aktivitäten an verschiedenen Zielen (Abb. 1). Um diese komplexe Alltagsmobilität zu bewältigen, braucht es Zeit, möglichst räumliche Nähe der Ziele oder eben den Zugang zu Verkehrsmitteln, die entfernt liegende Ziele erreichbar machen [5].

Für Menschen, die ohne eigenes Auto in ihren Wegeketten auf Verkehrsmittel des Umweltverbundes (Fuß- und Radverkehr, ÖPNV) oder die Kombination der Verkehrsmittel (Bus-Bus, Fahrrad-Bus etc.) angewiesen sind, sind die Verbindungssicherheit und Zuverlässigkeit der Verkehre unerlässlich. Hinzu kommt, dass Ziele bei der Verkehrs- und Mobilitätsplanung für Aufgaben der Care-Arbeit deutlich weniger berücksichtigt werden als Ziele für Erwerbsarbeit [6]. Die Abhängigkeit von Öffnungs- und Betriebszeiten (z. B. Kinderbetreuungseinrichtungen, Verwaltungsstellen) kommt hier erschwerend hinzu. Diese Realitäten ernst zu nehmen bedeutet, bei allen Entscheidungen zur Ausgestaltung unseres Verkehrssystems und unserer Umwelt die Auswirkungen auf Frauen und Männer in ihren sozialen Rollen mitzudenken und die daraus resultierenden Anforderungen zu berücksichtigen. Dies gilt unabhängig vom Alter der Menschen, denn die sozialen Rollen prägen das Leben auch bei älteren Frauen und Männern.

Unter Genderaspekten sind diese Bedingungen äußerst problematisch und erschweren eine selbstbestimmte Alltagsmobilität. Sie begünstigen Menschen mit dem Fokus Erwerbsarbeit und ohne Familienpflichten oder Mobilitätseinschränkungen sowie mit jederzeitiger Autoverfügbarkeit. Sie behindern Menschen mit einer vielfältigen Lebensrealität.

## Siedlungsstrukturelle Rahmenbedingungen von Mobilität im Alter

Über die an Ziele gebundenen Wegezwecke hinaus hat gerade die Mobilität im Alter eine wichtige subjektive Bedeutung. Zur Bedeutung von Mobilität im Alter gehören auch die Befriedigung grundsätzlicher Bedürfnisse wie die Bewegung an sich als menschliches physisches Bedürfnis, als Kontakt zur außerhäuslichen natürlichen Umwelt oder das Befriedigen des Bedürfnisses nach gesellschaftlicher Integration und Teilhabe. Ein weiterer nicht zu vernachlässigender Aspekt ist die Möglichkeit zur eigenständigen Fortbewegung als Ausdruck persönlicher Autonomie und Freiheit [7].

Mobilität wird durch verschiedene Rahmenbedingungen und Faktoren beeinflusst, die in engen Wechselwirkungen stehen. Auf der individuellen Ebene sind dies z. B. der ökonomische Status, das Alter oder Mobilitätseinschränkungen, die intersektional mit den geschlechtsspezifischen Aspekten verschränkt sind. Auf räumlicher Ebene sind dies die Stadt- und Siedlungsstruktur, die Nutzungsstruktur und Verteilung, d. h. die Verfügbarkeit von Angeboten (Versorgung und Dienstleistung), soziale Infrastruktur, Einrichtungen der Daseinsvorsorge und last, but not least der Zugang und die Verfügbarkeit von verkehrlicher Infrastruktur und deren Ausgestaltung. Die Alltagsmobilität aus Sicht einer älteren Person beschreibt folgender Absatz:

„Sind wohnungsnah keine Versorgungsangebote vorhanden, die ich zu Fuß oder mit dem Fahrrad gut erreichen kann, bin ich auf Verkehrsmittel angewiesen, die größere Entfernungen angemessen überwinden. Bin ich zu Fuß unterwegs und die Gehwege sind zugeparkt oder zu schmal für meinen Rollator, bin ich in meiner Mobilität zusätzlich eingeschränkt. Kann ich mir kein eigenes Auto leisten oder habe keinen Führerschein, bin ich darauf angewiesen, dass ich meine Erledigungen mit dem ÖPNV organisiere, der oft nur gelegentlich verkehrt. Oder ich bin nicht mehr unabhängig und auf Unterstützung z. B. durch Familienangehörige angewiesen. Hinzu kommt, dass ich mich davon abhalten lasse, einen abendlichen Termin wahrzunehmen, wenn ich mich im öffentlichen Raum oder in Bussen und Bahnen unsicher fühle.“

Fakt ist, dass die heutigen Siedlungs- und Nutzungsstrukturen die Alltagsmobilität erschweren. Denn Charakteristika unserer heutigen Stadt- und Raumstrukturen sind nicht räumliche Nähe und Nutzungsvielfalt, sondern weite Entfernungen oder Monostrukturen in Form von z. B. der lange Zeit in westdeutschen Bundesländern bevorzugten Ausweisung eines Baugebietes als ein reines Wohngebiet (WR). Dies wiederum hat zu einer Dominanz des Autoverkehrs im öffentlichen Raum geführt und hat die bevorrangte Zuweisung der Flächen für den fließenden und ruhenden Kfz-Verkehr zu Lasten des Fuß- und Radverkehrs zur Folge. Diese Entwicklung wird in den Regelwerken der Verkehrs- und Mobilitätsplanung weiter fest- und fortgeschrieben. So hat im Straßenverkehrsrecht in der Allgemeinen Verwaltungsvorschrift zur Straßenverkehrs-Ordnung (VwV-StVO) die Flüssigkeit und Leichtigkeit des Kfz-Verkehrs oberste Priorität für die Planung von Verkehrs- und Mobilitätsangeboten [8]. Auch bei der Radverkehrsplanung mit den Empfehlungen für Radverkehrsanlagen (ERA) oder den Richtlinien für die Anlage von Stadtstraßen (RASt) handelt es sich um technische Leitfäden. Nicht berücksichtigt werden dabei Elemente, die über die reine Vermeidung von Unfallschwerpunkten hinaus subjektive Unsicherheitsgefühle reduzieren und damit z. B. mehr Menschen das Radfahren ermöglichen [9].

## Daten und Fakten zur Mobilität im Alter aus Gendersicht

Wesentliche bundesweite Kenngrößen mit Genderrelevanz sind Führerscheinbesitz, Pkw-Besitz bzw. Verfügbarkeit, Verkehrsmittelzugang und -wahl, Wegezwecke und Distanzen. Daten hierzu sind den Ergebnissen der letzten bundesweiten Studie „Mobilität in Deutschland“ (MiD) zu entnehmen [10].

**Abb. 2:**
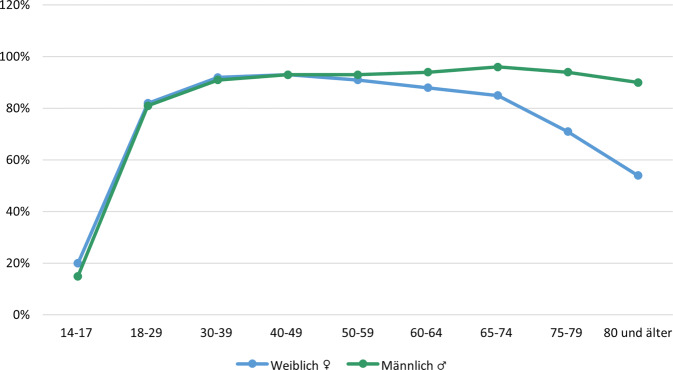
Pkw-Führerscheinbesitz nach Geschlecht und Alter 2017 (Quelle: eigene Abbildung nach Daten der Studie „Mobilität in Deutschland“ (MiD) in Tabellen http://www.mobilitaet-in-deutschland.de/MiT2017.html, abgerufen am 15.02.2024)

Danach sind die Unterschiede bei Führerscheinbesitz und Pkw-Verfügbarkeit im Alter bis 60 Jahre nicht signifikant. In den älteren Jahrgängen jedoch zeigte sich eine deutliche Diskrepanz zwischen den Geschlechtern: 54 % der Frauen, die älter als 80 Jahre waren, und 90 % der Männer, die älter als 80 Jahre waren, verfügten 2017 über einen Führerschein (Abb. 2).

Und auch wenn sich in den kommenden Jahren die Führerscheinbesitzquote weiter angleichen wird, werden die bestehenden ökonomischen Rahmenbedingungen gerade bei Seniorinnen kaum zu einer höheren Autoverfügbarkeit führen. Auch dies wird durch die Studie MiD belegt, wonach der Autobesitz in Haushalten mit niedrigen Einkommen bei weniger als 50 % liegt [10]. In der Gruppe mit niedrigem ökonomischen Status sind Seniorinnen nachweislich überrepräsentiert [11]. So lagen 2021 die Alterseinkünfte (einschließlich Hinterbliebenenrente und Privatvorsorge) von Frauen ab 65 mit rund 17.800 € 42 % unter den Alterseinkünften bei Männern mit rund 25.400 € [12]. Deutlich wird diese weibliche Mobilitätsarmut im Alter auch an der Tatsache, dass beim Übergang zur Wohnform Einpersonenhaushalt bei Seniorinnen häufiger auch ein Rückgang der Mobilität festzustellen ist [1].

**Abb. 3:**
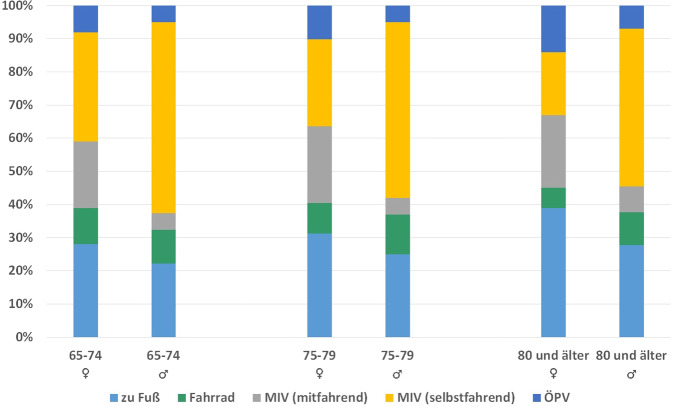
Verkehrsmittelwahl von Seniorinnen und Senioren nach Geschlecht und Alter 2017 (Quelle: eigene Abb. nach Daten der Studie „Mobilität in Deutschland“ (MiD) http://www.mobilitaet-in-deutschland.de/MiT2017.html, abgerufen am 15.02.2024). MIV: motorisierter Individualverkehr, ÖPV: beinhaltet den öffentlichen Nah- und Fernverkehr, inkl. Taxiverkehr

Daten zur Verkehrsmittelwahl und den zurückgelegten Distanzen aus MiD [10] zeigen, dass Seniorinnen eine deutlich höhere *Nahraumorientierung* haben als Senioren, d. h. täglich kürzere Strecken zurücklegen und dabei seltener selbst hinter dem Steuer sitzen. Sie sind eher zu Fuß, mit dem Fahrrad und mit öffentlichen Verkehrsmitteln oder als Beifahrerinnen unterwegs. Der Anteil der Seniorinnen, die häufiger zu Fuß unterwegs sind, ist trotz der höheren Anzahl hochaltriger Frauen in der Kohorte der älter als 80-Jährigen größer als der ihrer männlichen Altersgenossen. Hingegen ist die Fahrradnutzung bei den Seniorinnen und Senioren zwischen 65 und 74 nahezu gleich. Bei Senioren, die älter als 74 Jahre sind, ist die Fahrradnutzung höher als bei den gleichaltrigen Seniorinnen (Abb. 3). Gerade Seniorinnen zeigen öfter Unsicherheitsgefühle, wenn Fahrradwege zu schmal und/oder nicht vom Autoverkehr getrennt sind. Dadurch ist eine weitere Einschränkung der selbstbestimmten, von anderen Menschen unabhängigen Mobilität zu erwarten. Diese Befunde sind vor allem deshalb genderrelevant, weil die Zuständigkeit für Haus- und Versorgungsarbeit auch bei älteren Paaren oftmals bei den Frauen liegt und damit deren Mobilitätsbedarfe beeinflusst [2]. Dies wird z. B. an den Wegeketten deutlich, die auch bei älteren Frauen länger und differenzierter sind. So lagen bei der Untersuchung zu genderspezifischen Bedürfnissen von Mobilität im Kreis Stormarn der Anteil des Wegezweckes „Dienstleistung“ an allen Wegen der weiblichen Befragten älter als 67 Jahre mit 39 % deutlich über dem der männlichen Befragten älter als 67 Jahre mit 27 % [6].

Ein wichtiges Thema im Bereich Nahraumorientierung ist auch das Schaffen von *Möglichkeitsräumen zur Begegnung* und *Bewegung* außerhalb der eigenen Wohnung. Hochaltrige Frauen, die oftmals bereits verwitwet und damit alleinlebend sind, geben doppelt so häufig an, unter Einsamkeit zu leiden wie gleichaltrige Männer [13]. Daher sollten auch Mehrfachfunktionen der Wege und öffentlichen Räume dahin gehend ausgebaut werden, dass sie Aufenthalts- und Begegnungsmöglichkeiten (wieder) vermehrt und gesicherter anbieten. Gerade bei älteren Menschen und insbesondere bei den häufiger hochaltrig werdenden Frauen sind die klimatischen und wetterbedingten Aspekte wie Hitzeperioden, aber auch witterungsbedingte Risikofaktoren wie Glatteis oder Sichtbehinderung wichtige Faktoren, die die Entscheidung für Mobilität zusätzlich beeinflussen [1].

Es wird deutlich, wie wichtig eine gute Fußverkehrsinfrastruktur ohne die beschriebenen Hindernisse vor dem Hintergrund von Mobilitätseinschränkungen ist, die im Alter zunehmen: Während in der Gruppe der 60- bis 69-Jährigen etwa 16 % von Mobilitätseinschränkungen betroffen sind, steigt der Anteil auf über 50 % bei über 80-jährigen Frauen und Männern [14].

Das Thema „soziale Sicherheit“ prägt den Mobilitätsalltag vieler Seniorinnen und beinhaltet unter anderem auch die Gestaltung der gebauten Umwelt sowie den Zustand des öffentlichen Raums. Ältere Frauen beurteilen die soziale Sicherheit insbesondere abends und nachts aufgrund fehlender Beleuchtung, Unterführungen als einzige Wegeverbindung, Verschmutzung und Vandalismus, Abwesenheit von Menschen etc. kritischer und empfinden dies als eine Einschränkung in der Lebensqualität. Dies trifft auch auf die Nutzung des ÖPNV zu [15]. Das Gefühl für die soziale Sicherheit entscheidet, ob eine Seniorin ihre Wohnung noch verlässt oder lieber auf Teilhabe am gesellschaftlichen Leben verzichtet. Ältere Frauen wenden auch in anderen Bereichen sowohl als Fußgängerin wie auch als Autofahrerin häufiger als Männer Vermeidungsstrategien bei schlechten Wegeverhältnissen, unbekannten Gegenden oder Dunkelheit an [7]. Die im Allgemeinen schwierigere sozioökonomische Situation von Seniorinnen schränkt die Wahl gefühlt sicherer Verkehrsmittel wie Taxis oder eines eigenen Kfz zusätzlich ein.

## Anforderungen an eine gendersensible Mobilitätsplanung im Alter

Mobilität im Alter unter Gendergesichtspunkten zu betrachten bedeutet, die unterschiedlichen sozialen Rollen und geschlechtsspezifischen Rollenzuschreibungen sowie deren spezifische Auswirkungen für ältere Frauen und Männer in den Fokus zu stellen und die sich daraus ergebenden Anforderungen an die Verkehrssysteme, an Stadt- und Nutzungsstrukturen und an den öffentlichen Raum zu beachten, um gleichwertige Mobilitätschancen und gendergeprägte Mobilitätsbedarfe zu sichern. Dies erfordert eine integrierte Siedlungs- und Verkehrsplanung, orientiert am Leitbild der „Stadt der kurzen Wege“ und unter Berücksichtigung der individuellen Bedürfnisse. Konkret bedeutet das:

- *Integration einer gendersensitiven Mobilitätsplanung* von der Analyse mit geschlechtersegregierten Daten über die genderausgewogene Beteiligung der betroffenen Menschen bis hin zur Evaluierung der Wirkung und der Auswirkungen der Mobilitätsangebote auf die Mobilität von älteren Frauen und Männern;
- *Bereitstellung und Nutzung geschlechtersegregierter Daten*, die die spezifischen Alltags- und Mobilitätspraktiken älterer Frauen und Männer abbilden, bei der Mobilitätsplanung;
- *beteiligungsorientierte Planung* der Mobilitätsangebote, die die spezifischen Bedürfnisse und Mobilitätspraktiken älterer Frauen und Männer berücksichtigt;
- Einbeziehen der *Erreichbarkeit von Zie*len, die für ältere Frauen und Männer mit ihren Bedürfnissen und Aufgaben wichtig sind (z. B. Versorgung, Care-Arbeit, Beratung zu Pflege, Soziales, Gesundheit, Kultur und Sport, Begegnung) bei der Planung des öffentlichen Personennahverkehrs (ÖPNV) in städtischen und in ländlichen Räumen;
- durchgängige *Barrierefreiheit auch im ÖPNV*, z. B. kurze Einzugsbereiche von Haltepunkten, bauliche Barrierefreiheit, Informationen nach dem 2‑Sinne-Prinzip;
- *wohnungsnahe Ausstattung* mit Gütern und Dienstleistungen aller Art sowie mit sozialer Infrastruktur;
- ein *differenziertes Freizeit‑, Betreuungs- und Hilfsangebot* für Ältere unter Beachtung der höheren Lebenserwartung von Seniorinnen;
- *bauliche Anpassungen der verkehrlichen Fuß- und Radwegeinfrastruktur* wie ausreichend breite Gehwege, gesicherte, breite Radwege für unterschiedliche Geschwindigkeiten, abgesenkte Bordsteine und sichere Querungsangebote, verbunden mit der Schließung von Lücken im Fuß- und Radverkehrsnetz;
- *bauliche Anpassung von Flächen und Angeboten*, die für die Begleit- und Care-Mobilität von Bedeutung sind wie Bänke, Toiletten, (sichere) Abstellflächen für Rollatoren und Rollstühle;
- Ermöglichen der *eigenen aktiven Mobilität* zu jeder Tages- und Nachtzeit, z. B. durch Gewährleisten der sozialen Sicherheit, alternative Wegeführungen, Beleuchtung;
- Aufwertung des öffentlichen Raums als *Aufenthalts- und Bewegungsraum* durch Rastmöglichkeiten/Bänke, Toiletten, gute Beleuchtung;
- *Überwachung des ruhenden Kfz-Verkehrs* und konsequente Ahndung von Parken auf Geh- und Radwegen sowie vor den Gehwegabsenkungen;
- Berücksichtigung von *klimatischen Aspekten für die Alltagsmobilität und Nahraumorientierung,* wie z. B. Hitze (z. B. Beschattungen im öffentlichen Raum, Wasserspender), aber auch das Sichern von Wegebeziehungen z. B. bei Glatteis und Schnee;
- Bereitstellung *flexibler und kostengünstiger Angebote* im ländlichen Raum als Teil des ÖPNV oder als Teil kommunaler Konzepte;
- Bereitstellung *multimodaler Sharing-Angebote*, um die Abhängigkeit vom Besitz eines eigenen Autos zu minimieren bzw. Alternativen anzubieten.

## Fazit

Unabhängig davon, dass das Auto bei der Mobilität im Alter weiterhin eine Rolle spielen wird und auch Seniorinnen bei steigender Lebenserwartung zunehmend selbstverständlicher einen Führerschein besitzen und Autos nutzen, ist der Förderung des Umweltverbundes (Fuß- und Radverkehr, ÖPNV) Priorität einzuräumen und damit ein Beitrag zu leisten, die Dominanz des fließenden und ruhenden Kfz-Verkehrs in der Mobilitätsplanung zu brechen. Ziel ist es, das Alter für Frauen und Männer in einer lebenswerten Umgebung zu gestalten und ihnen zu ermöglichen, am gesellschaftlichen Leben aktiv teilzunehmen.

Deutlich mehr als bisher ist bei der Planung und Umsetzung von Mobilitätsmaßnahmen in der vorbereitenden Analyse, in der Zielsetzung, im Planungs- und Umsetzungsprozess die Vielfalt der Lebenssituationen Älterer und insbesondere älterer Frauen von vornherein systematisch zu berücksichtigen. Die Wirkung und Folgen der Mobilitätsmaßnahmen sind zu evaluieren.

Auch wenn im Alter die Mobilität allmählich abnimmt und oft durch körperliche Einschränkungen geprägt ist, müssen ältere Menschen in einer eigenständigen, aktiven Mobilität unterstützt werden und das nicht nur aus Gründen der Gesundheitsvorsorge, sondern auch zur Sicherstellung ihrer Teilhabe am gesellschaftlichen Leben. Aufgrund der strukturellen und gesellschaftlichen immer noch bestehenden geschlechterspezifischen Ungleichheiten ist dabei gerade im Alter die Einbeziehung der Genderperspektive bei Mobilitätsplanungen von Anfang an unerlässlich.

## Footnotes

1 Die Autorinnen verwenden hier zur Verdeutlichung die binären Bezeichnungen „Frauen“ und „Männer“, da die Rollenzuschreibungen und ihre Umsetzung in der baulichen und verkehrlichen Infrastruktur (noch) überwiegend entlang dieser geschlechtlichen Zuordnung erfolgen. Damit ist keineswegs eine Nichtberücksichtigung von LGBTQ+ und anderen Geschlechtsidentitäten verbunden.
